# Indole derivatives intervene osteoporosis via gut-bone axis

**DOI:** 10.3389/fcimb.2026.1845772

**Published:** 2026-08-20

**Authors:** Wendong Wang

**Affiliations:** Department of Engineering Mechanics, Dalian University of Technology, Dalian, China

**Keywords:** gut-bone axis, indole derivatives, molecular mechanism, osteoporosis, translational prospects

## Abstract

Osteoporosis is a systemic skeletal disease characterized by low bone mass, deterioration of bone microarchitecture, and increased fracture risk, which imposes a heavy burden on global public health, especially in postmenopausal women and the elderly. The gut-bone axis, a bidirectional regulatory network between the gut microbiota and bone metabolism, has emerged as a novel therapeutic target for osteoporosis. Indole derivatives, a class of bioactive compounds derived from tryptophan metabolism by gut microbiota or plant secondary metabolism, have attracted increasing attention due to their extensive biological activities, including anti-inflammatory, antioxidant, and metabolic regulatory effects. Accumulating evidence indicates that indole derivatives can regulate bone homeostasis through the gut-bone axis, providing a new strategy for the prevention and treatment of osteoporosis. This review systematically summarizes the types of indole derivative, elaborates their molecular mechanisms in the treatment of osteoporosis via the gut-bone axis (including regulating gut microbiota composition, maintaining intestinal barrier integrity, modulating immune-inflammatory responses, and mediating key signaling pathways), and discusses the current translational progress, existing challenges, and future prospects. This review aims to provide a comprehensive theoretical basis for the clinical application of indole derivatives in osteoporosis treatment and the development of novel targeted drugs.

## 1 Introduction

Osteoporosis, a prevalent age-related metabolic bone disorder, is defined by the World Health Organization (WHO) as a condition of compromised bone mineral density (BMD) and deteriorated bone microarchitecture, which consequently increases bone fragility and susceptibility to fracture (Morin et al., 2025). This disease causes approximately 9 million fractures every year worldwide, including spine fractures, hip fractures, wrist fractures, humerus fractures, and pelvis fractures (Fujiwara et al., 2022). Hip fractures cause major morbidity, mortality and long-term disability among older persons worldwide (Chandran et al., 2026). The global demographic shift toward an aging population has led to a markedly increased osteoporosis prevalence, causing a serious public health burden (Fuggle et al., 2025). Current pharmacological interventions for osteoporosis primarily comprise antiresorptive agents (such as bisphosphonates, and denosumab) and anabolic agents (such as teriparatide). However, their clinical utility is often constrained by adverse effects, long-term safety concerns, and suboptimal efficacy in a subset of patients (Foessl et al., 2023). Consequently, there is an urgent and compelling need to develop novel, safe, and more effective therapeutic strategies for the management of osteoporosis.

The concept of the gut-bone axis, describes a bidirectional communication network between the gut microbiota and the skeletal system, mediated by microbial metabolites, immune mediators, endocrine signals, and nutrient absorption (Indrio and Salatto, 2025). Acting as a “virtual endocrine organ”, the gut microbiota plays a pivotal role in maintaining skeletal homeostasis by orchestrating the delicate balance between osteoblast-mediated bone formation and osteoclast-mediated bone resorption (Ticinesi et al., 2025). Disruption of gut microbial homeostasis can compromise intestinal barrier integrity, trigger aberrant immune-inflammatory responses, and impair nutrient absorption—all of which are closely implicated in the pathogenesis of osteoporosis (Li and Guo, 2026). For instance, in an ovariectomized (OVX) estrogen deficiency-induced osteoporosis mouse model, alterations in the gut microbiota and decreases in levels of tryptophan metabolites have been observed, decreased abundance of *Lactobacillus* and *Clostridium* in the gut and reduced serum levels of indoleacrylic acid (IA), indoleacetic acid, and indolepropionic acid (Bai et al., 2026).

Indole derivatives constitute a class of heterocyclic compounds characterized by an indole ring structure, which are ubiquitously distributed in nature. These compounds can be broadly categorized into two major groups: microbial indole derivatives—such as indole-3-lactic acid (ILA), indole-3-propionic acid (IPA), and indole-3-acetic acid (IAA)—which are generated via gut microbiota-mediated tryptophan metabolism (Jia et al., 2024), and plant-derived indole derivatives, including indole alkaloids such as vindoline, rutaecarpine, and harmine (Zhang et al., 2022; Li et al., 2023; Zhai et al., 2025). In recent years, a number of studies have demonstrated that indole derivatives exert significant modulatory effects on bone metabolism, with mechanisms closely linked to the gut-bone axis. For example, IA supplementation has been shown to protect against OVX-induced bone loss in mice by suppressing osteoclastogenesis (Bai et al., 2026), while IAA and IPA enhance gut barrier integrity and promote skeletal health through activation of the aryl hydrocarbon receptor (AHR) signaling pathway (Chen et al., 2024). Despite these advances, a systematic synthesis of the molecular mechanisms and translational prospects of indole derivatives in osteoporosis therapy via the gut-bone axis remains conspicuously absent. This review integrates the latest high-quality findings from multiple databases to comprehensively elucidate the role of indole derivatives within the gut-bone axis and their underlying mechanisms including gut microbiota modulation, intestinal barrier integrity preservation, immune-inflammatory responses regulation, and key signaling pathways mediation in the treatment of osteoporosis. Meanwhile, this review also critically evaluating their current translational progress, existing challenges, limitations, and future perspective. By bridging the gaps between fundamental mechanistic understanding and translational application, and by focusing on indole derivatives as a distinct class of compounds specifically, this review provides a unique and novel framework that complements and extends the existing literature, offering actionable insights for both basic researchers and clinicians interested in harnessing the gut-bone axis for osteoporosis management.

## 2 Indole derivatives involved in bone metabolism regulation

Indole derivatives implicated in the regulation of the gut-bone axis originate primarily from two sources: gut microbiota metabolism and plant secondary metabolism. From a chemical and origin perspective, microbial derivatives (IA, ILA, IPA, and IAA) are relatively simple, short-chain tryptophan catabolites with modest molecular weights and polar side chains, whereas plant alkaloids (vindoline, rutaecarpine, and harmine) possess complex, rigid polycyclic architectures that confer different physicochemical properties. Despite their structural diversity and distinct biological activities, these compounds converge in their capacity to modulate the gut-bone axis, thereby influencing bone homeostasis through different action mechanisms and signaling pathways. The principal categories of these indole derivatives and their respective sources are delineated as follows.

### 2.1 Microbial-derived indole derivatives

Microbial-derived indole derivatives represent the primary products of tryptophan metabolism by gut microbiota—including commensal genera such as *Bacteroides*, *Clostridium*, *Lactobacillus*, and *Bifidobacterium*—and constitute a substantial proportion of the indole derivatives present in the human body (Roager and Licht, 2018). Tryptophan, an essential amino acid, undergoes microbial metabolism via the indole pathway to generate a diverse array of indole derivatives, including IA, IAA, IPA, indole-3-aldehyde, and indole-3-acetaldehyde (Agus et al., 2018). Specific bacterial species and key enzymes are responsible for the biosynthesis of distinct indole metabolites, and the composition of the gut microbiota thus critically determines the profile of indole derivatives produced. For example, in the production pathway of ILA from tryptophan catabolism in *Bifidobacterium longum* subsp. *infantis*, tryptophan is converted to indole-3-pyruvate by the amino acid transaminase (*aat*), which is subsequently reduced to ILA by aromatic lactate dehydrogenase (*aldh*) (Zhang et al., 2025b). In the IPA producer *Clostridium sporogenes*, key enzymes including the phenyllactate dehydrogenase (*fldH*; indole-3-pyruvate to ILA conversion), the phenyllactate dehydratase enzyme complex (*fldLAIBC*; ILA to indole-3-acrylate conversion) and the acyl-CoA dehydrogenase (*acdA*; indole-3-acrylate to IPA conversion) have been identified specifically (Zund et al., 2025). These metabolites are absorbed across the intestinal barrier into the systemic circulation and subsequently transported to the skeletal system, where they exert modulatory effects on bone metabolism through the gut-bone axis (Xiang et al., 2025).

Among microbial-derived indole derivatives, IA, IAA, and IPA represent the most extensively investigated candidates in the context of bone health. A study demonstrated that serum IA levels are significantly reduced in OVX-induced osteoporosis mouse model, with IA levels exhibiting a positive correlation with bone mass (Bai et al., 2026). IAA and IPA, both serving as ligands for the AHR, activate AHR signaling within intestinal epithelial cells, thereby enhancing intestinal barrier integrity and modulating immune-inflammatory responses—processes that are intimately linked to skeletal homeostasis (Chen et al., 2024). Additionally, other tryptophan-derived metabolites, including indole-3-ethanol, indole-3-pyruvate, and indole-3-aldehyde, have been shown to enhance intestinal barrier function by modulating components of the apical junctional complex, such as myosin IIA and ezrin, while concurrently reducing inflammatory responses, further contributing to the regulation of the gut-bone axis (Wei et al., 2025).

### 2.2 Plant-derived indole derivatives

Plant-derived indole derivatives primarily comprise indole alkaloids, which serve as secondary metabolites synthesized by various plant families, including *Rutaceae*, *Apocynaceae*, and *Leguminosae* (Oladeji et al., 2024). These compounds are characterized by distinct chemical architectures and a broad spectrum of biological activities, with a subset demonstrated to modulate bone metabolism. Representative plant-derived indole derivatives implicated in the regulation of bone metabolism include vindoline, rutaecarpine, harmine, and their structural analogs (Caruso et al., 2024). Specifically, *in vivo* investigation in vindoline-treated mice has demonstrated protection against trabecular bone degradation and bone loss induced by OVX (Zhan et al., 2019). The potential of rutaecarpine to reduce the age-related effects of human bone marrow stromal stem cells (hBMSCs) has also been investigated. The results revealed that rutaecarpine enhanced osteoblastic differentiation of hBMSCs and reduced the burden of cellular senescence and inflammation, suggesting a possible role for rutaecarpine as small molecule antioxidant agents that can be used to prevent impaired osteoblastic functions and bone loss associated with aging and osteoporosis (Ali et al., 2024). Similarly, oral administration of harmine emulsion to OVX mice resulted in enhanced trabecular bone mass and osteogenic responses, increased numbers of preosteoclasts, as well as reduced numbers of osteoclasts and fat cells (Huang et al., 2018). Collectively, these plant-derived indole derivatives offer promising candidates for the development of novel osteoporosis therapeutics that target bone metabolism. However, there are still limitations in the research on the mechanisms by which plant-derived indole derivatives improve bone metabolism via the gut-bone axis.

## 3 Mechanisms of indole derivatives treating osteoporosis via the gut-bone axis

**Figure 1:**
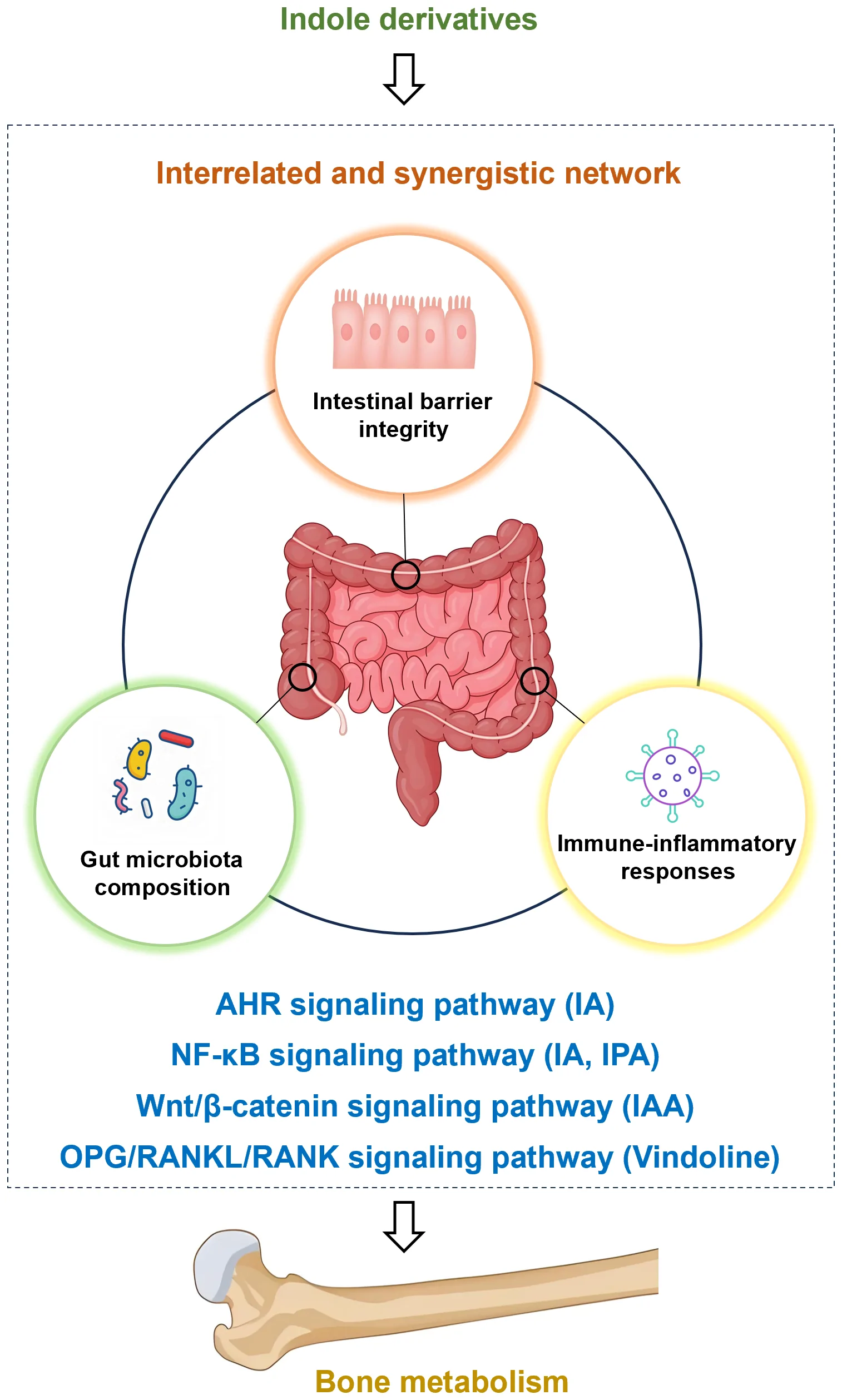
Mechanisms of indole derivatives regulating bone metabolism through gut-bone axis.

Indole derivatives regulate bone metabolism through multiple molecular mechanisms via the gut-bone axis, mainly including regulating gut microbiota composition, maintaining intestinal barrier integrity, modulating immune-inflammatory responses, and mediating key signaling pathways. These mechanisms are interrelated and synergistic, forming a complex regulatory network (**Figure 1**). Specifically, indole derivatives initially regulate gut microbiota composition, which subsequently strengthens intestinal barrier integrity. An intact barrier prevents the translocation of pro-inflammatory microbial products, thereby modulating immune-inflammatory responses. The consequent anti-inflammatory microenvironment, together with the direct actions of indole derivatives, ultimately orchestrates the key signaling pathways involved in bone metabolism. This hierarchical and synergistic network ensures that local alterations in the gut effectively translate into protective effects on bone metabolism.

### 3.1 Regulating gut microbiota composition

Gut microbiota dysbiosis is an important factor leading to osteoporosis, which is characterized by a decrease in beneficial bacteria and an increase in harmful bacteria (Liu et al., 2026). Indole derivatives have been shown to modulate gut microbiota composition and restore microbial homeostasis, thereby exerting beneficial effects on bone metabolism. For example, a study found that supplementation with IAA and IPA effectively remodeled the gut microbiota of OVX mice, increased the abundance of *Lactobacillus* and *Bifidobacterium*, and reduce the abundance of harmful bacteria, thereby ameliorating OVX-induced bone loss (Chen et al., 2024). The mechanisms by which indole derivatives modulate gut microbiota composition are multifaceted, involving the suppression of pathogenic bacterial growth and the promotion of beneficial bacterial proliferation. For example, ILA has been shown to effectively inhibit foodborne pathogens such as *Salmonella* spp., *Staphylococcus* spp., *Escherichia coli* and *Listeria monocytogene*, while significantly stimulate the growth of many bacteria known to promote human health, including the genera *Bifidobacterium*, *Faecalibacterium*, *Dislister* and *Dore* (Zhou et al., 2022). Furthermore, indole derivatives can modulate the expression of microbial genes involved in tryptophan metabolism, thereby enhancing the production of beneficial metabolites and contributing to the maintenance of gut microbiota homeostasis (Yong et al., 2024).

### 3.2 Maintaining intestinal barrier integrity

The intestinal barrier constitutes a critical component of the gut-bone axis, functioning to prevent the translocation of intestinal endotoxins—such as lipopolysaccharide (LPS)—and other harmful metabolites into the systemic circulation, thereby avoiding systemic inflammation and subsequent disruption of bone metabolism (Li and Guo, 2026; Kayama et al., 2020). Intestinal barrier dysfunction, which is frequently observed in both osteoporosis patients and relevant animal models, is closely linked to gut microbiota dysbiosis (Chen et al., 2025). Indole derivatives contribute to the preservation of intestinal barrier integrity through multiple mechanisms, including the upregulation of tight junction protein, enhancement of intestinal epithelial cell proliferation, and attenuation of intestinal inflammation (Li et al., 2021). Recent studies demonstrate that IAA and IPA activate AHR signaling in intestinal epithelial cells, and promote epithelial regeneration and accelerates intestinal barrier repair (Chen et al., 2024; Zhang et al., 2025a). Additionally, other tryptophan-derived metabolites, including indole-3-ethanol, indole-3-pyruvate, and indole-3-aldehyde, have been shown to modulate the expression of the apical junctional complex while concurrently reducing intestinal inflammation, thereby enhancing intestinal barrier function (Wei et al., 2025). Maintenance of an intact intestinal barrier effectively inhibits the translocation of LPS and other harmful metabolites, thereby dampening systemic inflammatory responses and preserving bone homeostasis (Rios-Arce et al., 2017; Gong et al., 2025).

### 3.3 Modulating immune-inflammatory responses

Chronic low-grade inflammation represents a key pathological feature of osteoporosis, which can promote osteoclast differentiation while inhibit osteoblast proliferation, leading to bone loss (Livshits and Kalinkovich, 2022). As the largest immune organ in the body, the gut harbors a complex microbiota whose metabolites exert profound regulatory effects on the immune system, subsequently modulating systemic inflammatory responses (Zeng et al., 2025). Through the gut-bone axis, indole derivatives are capable of modulating immune-inflammatory responses, thereby exerting beneficial effects on bone metabolism (Xu et al., 2025). The immunomodulatory actions of indole derivatives are primarily mediated through two complementary mechanisms. First, they promote the polarization of M2 macrophages—characterized by an anti-inflammatory phenotype—and enhance the production of anti-inflammatory cytokines such as interleukin-10 (IL-10), while concurrently suppressing the release of pro-inflammatory cytokines including tumor necrosis factor-α (TNF-α) and interleukin-6 (IL-6) (Chen et al., 2024). Second, they inhibit the activation of T helper 17 (Th17) cells and facilitate the differentiation of regulatory T (Treg) cells, thereby contributing to the maintenance of immune homeostasis (Su et al., 2024). For instance, IA has been shown to suppress the expression of the pro-inflammatory cytokines TNF-α and IL-6 in bone marrow macrophages (BMMs) and to reduce the nuclear translocation of phosphorylated p65, a key event in NF-κB signaling, thereby inhibiting osteoclast resorptive activity (Bai et al., 2026). Furthermore, microbial-derived indole derivatives have been shown to be involved in suppressing arthritis by activating AHR in regulatory B cells (Rosser et al., 2020), suggesting a potential mechanistic link between gut microbial indole derivatives, B cell function, and skeletal pathology. However, direct evidence connecting specific indole metabolites to B cell-mediated bone regulation is lacking. Collectively, these immunomodulatory effects of indole derivatives serve to effectively alleviate the bone loss associated with chronic inflammation.

### 3.4 Mediating key signaling pathways in bone metabolism

Through the gut-bone axis, indole derivatives can mediate key signaling pathways in bone metabolism, thereby regulating the balance between osteoblast-mediated bone formation and osteoclast-mediated bone resorption. The main signaling pathways involved in the processes include the AHR signaling pathway, the NF-κB signaling pathway, the Wnt/β-catenin signaling pathway, and the OPG/RANKL/RANK signaling pathway (**Table 1**).

**Table 1:** Key signaling pathways of indole derivatives for regulating bone metabolism.

Indole derivatives	Receptor involved	Target cell type	Experimental model	Principal biological outcome	Ref.
IA	AHR	BMMs	*In vitro*: RANKL-stimulated BMMs	Inhibits RANKL-induced AHR and c-Fos expression while reducing the nuclear translocation of phosphorylated p65, thereby ultimately suppressing osteoclastogenesis.	(Bai et al., 2026)
IA	NF-κB	BMMs	*In vitro*: RANKL-stimulated BMMs	Downregulates the expression of NF-κB target genes (such as MMP-9, TRAP), and ultimately inhibits osteoclast resorptive activity.	(Bai et al., 2026)
IPA	NF-κB	Osteoclasts	*In vivo*: High-fat-diet induced obese mice	Inhibits NF-κB/NLRP3-mediated osteoclastogenesis and improves bone quality.	(Wu et al., 2025)
IAA	AHR	Caco‐2; Osteoblasts	*In vivo*: OVX mice; *In vitro*: LPS‐treated Caco‐2 cells	Activates AHR signaling in intestinal epithelial cells, leading to the stimulation of the Wnt/β-catenin signaling pathway, which not only promotes intestinal epithelial regeneration but also promotes osteoblast differentiation and bone formation.	(Chen et al., 2024)
Vindoline	RANKL/RANK	BMMs	*In vivo*: OVX mice; *In vitro*: RANKL-stimulated BMMs	Inhibits RANKL-induced osteoclastogenesis and prevents ovariectomy-induced bone loss in mice.	(Zhan et al., 2019)

The AHR signaling pathway represents a key mediator of the regulatory effects exerted by indole derivatives on the gut-bone axis. AHR is a ligand-activated transcription factor widely expressed in intestinal epithelial cells, immune cells, osteoblasts, and osteoclasts (Kim et al., 2024). Indole derivatives—including IA, IAA, and IPA—can bind to AHR, activating downstream signaling and subsequently modulating bone metabolism (Chen et al., 2024; Bai et al., 2026; Xu et al., 2025). For instance, IA has been shown to inhibit RANKL-induced AHR and c-Fos expression while reducing the nuclear translocation of phosphorylated p65, thereby ultimately suppressing osteoclastogenesis (Bai et al., 2026). Conversely, AHR activation has also been demonstrated to promote osteoblast proliferation and differentiation through the regulation of osteogenic genes such as *Runx2* and *Ocn* (Huang et al., 2023).

The NF-κB signaling pathway plays a central role in osteoblast differentiation, osteoclast differentiation, and the regulation of inflammatory responses (Boyce et al., 2023). Indole derivatives have been shown to inhibit NF-κB pathway activation, thereby suppressing osteoclastogenesis and attenuating bone resorption. For instance, IA reduces the nuclear translocation of phosphorylated p65 in BMMs, downregulates the expression of NF-κB target genes (such as MMP-9, TRAP), and ultimately inhibits osteoclast resorptive activity (Bai et al., 2026). IPA inhibits NF-κB/NLRP3-mediated osteoclastogenesis and improves bone quality in high-fat-diet induced obese mice (Wu et al., 2025).

The Wnt/β-catenin signaling pathway plays a pivotal role in promoting osteoblast proliferation and differentiation (Wrobel et al., 2025; Wang et al., 2024). Through the gut-bone axis, indole derivatives can activate the Wnt/β-catenin signaling pathway, thereby enhancing bone formation. For example, IAA activates AHR signaling in intestinal epithelial cells, leading to the stimulation of the Wnt/β-catenin signaling pathway, which not only promotes intestinal epithelial regeneration but also promotes osteoblast differentiation and bone formation (Chen et al., 2024).

The OPG/RANKL/RANK signaling pathway represents the core pathway regulating osteoclast differentiation. The binding of RANKL to its cognate receptor RANK on the surface of osteoclast precursor cells triggers osteoclast differentiation and activation, whereas OPG can bind to RANKL, thereby inhibiting the RANKL-RANK interaction and suppressing osteoclastogenesis (Edwards and Mundy, 2011). Indole derivatives can regulate the OPG/RANKL/RANK signaling pathway by modulating gut microbiota and immune factors, thereby maintaining the balance of bone metabolism. For instance, vindoline inhibits RANKL-induced osteoclastogenesis and prevents ovariectomy-induced bone loss in mice (Zhan et al., 2019). However, current scarcity of experimental evidence for this pathway is a limitation of the research.

## 4 Translational prospects and challenges of indole derivatives in osteoporosis treatment

With the deepening understanding of the molecular mechanisms by which indole derivatives exert their therapeutic effects on osteoporosis via the gut-bone axis, their translational potential has garnered increasing attention. To date, indole derivatives have demonstrated favorable therapeutic efficacy in preclinical studies, with emerging progress also being made in clinical investigations. Nevertheless, several challenges remain to be addressed prior to their widespread clinical adoption. The following sections discuss the current translational progress, existing challenges, and future perspectives regarding indole derivatives in this context.

Preclinical studies have confirmed the therapeutic efficacy of various indole derivatives in the management of osteoporosis. For instance, supplementation with IA in OVX-induced osteoporotic mice has been shown to protect against bone loss, accompanied by elevated levels of PINP (a marker of bone formation), and reduced levels of CTX-1 (a marker of bone resorption) (Bai et al., 2026). Supplementation with IAA and IPA has also been demonstrated to ameliorate OVX-induced bone loss by restoring the intestinal AHR-mediated gut-bone signaling axis (Chen et al., 2024). Furthermore, plant-derived indole derivatives, including rutaecarpine and harmine, have exhibited favorable anti-osteoporotic effects in animal models (Tsamo et al., 2019). With regard to clinical trials, while dedicated clinical studies evaluating the efficacy of indole derivatives for osteoporosis remain limited, emerging evidence from related investigations has underscored their therapeutic potential. For instance, a clinical study reported that serum levels of IPA are positively correlated with BMD in postmenopausal women, suggesting that IPA may serve as a potential biomarker and therapeutic target for osteoporosis (Qiu et al., 2026). Another clinical study demonstrated that tryptophan supplementation—a precursor of indole derivatives—can increase the level of indole derivatives in the gut, improve gut microbiota composition, and have a positive effect on bone health in the elderly (Qiu et al., 2025). Collectively, these findings provide a basis for the clinical application of indole derivatives in osteoporosis treatment.

Despite the promising preclinical efficacy of indole derivatives, several challenges remain to be addressed prior to their clinical translation. First, the bioavailability of indole derivatives is suboptimal. Most of these compounds exhibit poor water solubility and are susceptible to degradation within the gastrointestinal tract, resulting in low absorption efficiency and consequently limited therapeutic efficacy (Matteo et al., 2026; Shukla et al., 2025). Second, the safety of indole derivatives requires further validation. Although preclinical studies have indicated favorable biocompatibility and low toxicity, their long-term safety in humans (such as potential adverse effects, drug interactions) remains to be elucidated (Wang et al., 2026). Third, the specific dosage and administration route of indole derivatives for osteoporosis treatment have not been determined. Given that different indole derivatives possess distinct biological activities and pharmacokinetic properties, in-depth pharmacokinetic studies are warranted to determine the optimal dosage and administration route. Fourth, the molecular mechanism of indole derivatives in the treatment of osteoporosis via the gut-bone axis remain incompletely understood. Although some key signaling pathways have been identified, the complex regulatory network connecting indole derivatives, gut microbiota, and bone metabolism still needs further investigation.

Beyond these translational challenges, a critical appraisal of the existing evidence reveals methodological limitations, interpretative inconsistencies, and knowledge gaps. First, most preclinical studies are confined to the OVX mouse model, which poorly recapitulates the multifactorial, age-related pathophysiology of human osteoporosis. Moreover, nearly all efficacy studies employ prophylactic rather than therapeutic dosing regimens. Second, conflicting findings are often ignored. For instance, AHR activation by IAA and IPA is generally considered beneficial, yet AHR signaling is highly context-dependent and can paradoxically promote pro-inflammatory Th17 responses while suppressing Treg differentiation, potentially aggravating bone loss depending on the host’s inflammatory status. Third, the gaps between *in vitro*, animal, and clinical evidence are obvious. The concentrations effective in cell culture and animal models are far exceed the levels detected in human circulation, brings challenges for exploring the optimal dosage and administration route, and safety of indole derivatives in osteoporosis patients. Furthermore, the compositional differences between animal and human gut microbiota preclude linear extrapolation of animal data to human. Fourth, major knowledge gaps persist, including the drug-drug interactions, the influence of sex, age, and diet on endogenous metabolite production.

To promote the clinical translation of indole derivatives for the treatment of osteoporosis, the following directions can be focused on in the future. First, improve the bioavailability of indole derivatives. Novel drug delivery systems (such as nanocarriers, liposomes, microspheres) can be used to enhance the water solubility and stability of indole derivatives, thereby improving their absorption efficiency and prolonging their half-life (Shukla et al., 2025; Czapka et al., 2022). Second, conduct more large-scale, long-term clinical trials to verify the safety and efficacy of indole derivatives in osteoporosis patients, and determine the optimal dosage and administration route. Third, further explore the molecular mechanisms of indole derivatives in the treatment of osteoporosis via the gut-bone axis, identify new targets and signaling pathways, and provide a theoretical basis for the development of novel indole derivative-based drugs. Fourth, develop combined therapies. Indole derivatives can be combined with existing anti-osteoporosis drugs (such as bisphosphonates, teriparatide) to improve therapeutic efficacy and reduce adverse effects (Singh et al., 2008). Fifth, explore the potential of indole derivatives as biomarkers for osteoporosis. Since the levels of some indole derivatives (such as IPA) are closely related to BMD, they can be developed as biomarkers for the diagnosis and prognosis of osteoporosis (Qiu et al., 2026).

## 5 Conclusion

Osteoporosis is a major global public health problem, and the gut-bone axis has become a novel therapeutic target for this disease. Indole derivatives, as bioactive compounds derived from gut microbiota and plants, can regulate bone homeostasis through multiple molecular mechanisms via the gut-bone axis, including regulating gut microbiota composition, maintaining intestinal barrier integrity, modulating immune-inflammatory responses, and mediating key signaling pathways (AHR, NF-κB, Wnt/β-catenin, OPG/RANKL/RANK). Preclinical studies have shown that indole derivatives have good anti-osteoporosis effects, and some clinical studies have confirmed their potential in bone health regulation. However, there are still some challenges in their translational application, such as low bioavailability, unclear long-term safety, and undetermined optimal dosage. In the future, with the continuous advancement of research, the development of novel drug delivery systems, and the conduct of more in-depth clinical trials, indole derivatives are expected to become a new class of anti-osteoporosis drugs targeting the gut-bone axis, providing a safe, effective, and novel therapeutic strategy for the prevention and treatment of osteoporosis. At the same time, further exploration of the molecular mechanisms of indole derivatives in the gut-bone axis will help to deepen the understanding of the interaction between gut microbiota and bone metabolism, and provide new ideas for the treatment of other bone-related diseases.
